# *Leishmania enriettii*: biochemical characterisation of lipophosphoglycans (LPGs) and glycoinositolphospholipids (GIPLs) and infectivity to *Cavia porcellus*

**DOI:** 10.1186/s13071-015-0633-8

**Published:** 2015-01-17

**Authors:** Larissa Ferreira Paranaíba, Rafael Ramiro de Assis, Paula Monalisa Nogueira, Ana Claúdia Torrecilhas, João Henrique Campos, Amanda Cardoso de Oliveira Silveira, Olindo Assis Martins-Filho, Natalia Lima Pessoa, Marco Antônio Campos, Patrícia Martins Parreiras, Maria Norma Melo, Nelder de Figueiredo Gontijo, Rodrigo Pedro Pinto Soares

**Affiliations:** Centro de Pesquisas René Rachou, Fundação Oswaldo Cruz, FIOCRUZ, Belo Horizonte, Minas Gerais Brazil; Laboratório de Fisiologia de Insetos Hematófagos, Departamento de Parasitologia, Instituto de Ciências Biológicas, Universidade Federal de Minas Gerais, Belo Horizonte, MG Brazil; Laboratório de Biologia de Leishmania, Departamento de Parasitologia, Instituto de Ciências Biológicas, Universidade Federal de Minas Gerais, Belo Horizonte, Brazil; Laboratório de Imunologia Celular e Bioquímica de Fungos e Protozoários, Departamento de Ciências Biológicas, Campus Diadema, Universidade Federal de São Paulo, UNIFESP, São Paulo, SP Brazil; Laboratory of Cellular and Molecular Parasitology, Centro de Pesquisas René Rachou, Fundação Oswaldo Cruz, FIOCRUZ, Av. Augusto de Lima 1715, Belo Horizonte, Minas Gerais 30190-002 Brazil

**Keywords:** *Leishmania enriettii*, Glycoconjugates, Lipophosphoglycan, Glycoinositolphospholipids, *Cavia porcellus*, Macrophage, Innate immunity

## Abstract

**Background:**

*Leishmania enriettii* is a species non-infectious to man, whose reservoir is the guinea pig *Cavia porcellus*. Many aspects of the parasite-host interaction in this model are unknown, especially those involving parasite surface molecules. While lipophosphoglycans (LPGs) and glycoinositolphospholipids (GIPLs) of *Leishmania* species from the Old and New World have already been described, glycoconjugates of *L. enriettii* and their importance are still unknown.

**Methods:**

Mice peritoneal macrophages from C57BL/6 and knock-out (TLR2 −/−, TLR4 −/−) were primed with IFN-γ and stimulated with purified LPG and GIPLs from both species. Nitric oxide and cytokine production were performed. MAPKs (p38 and JNK) and NF-kB activation were evaluated in J774.1 macrophages and CHO cells, respectively.

**Results:**

LPGs were extracted, purified and analysed by western-blot, showing that LPG from L88 strain was longer than that of Cobaia strain. LPGs and GIPLs were depolymerised and their sugar content was determined. LPGs from both strains did not present side chains, having the common disaccharide Gal(β1,4)Man(α1)-PO_4_. The GIPL from L88 strain presented galactose in its structure, suggestive of type II GIPL. On the other hand, the GIPL of Cobaia strain presented an abundance of glucose, a characteristic not previously observed. Mice peritoneal macrophages from C57BL/6 and knock-outs (TLR2 -/- and TLR4 -/-) were primed with IFN-γ and stimulated with glycoconjugates and live parasites. No activation of NO or cytokines was observed with live parasites. On the other hand, LPGs and GIPLs were able to activate the production of NO, IL-6, IL-12 and TNF–α preferably via TRL2. However, in CHO cells, only GIPLs were able to activate TRL2 and TRL4. *In vivo* studies using male guinea pigs (*Cavia porcellus*) showed that only strain L88 was able to develop more severe ulcerated lesions especially in the presence of salivary gland extract (SGE).

**Conclusion:**

The two *L. enriettii* strains exhibited polymorphisms in their LPGs and GIPLs and those features may be related to a more pro-inflammatory profile in the L88 strain.

## Background

The leishmaniases are diseases caused by the protozoa *Leishmania* (Kinetoplastida, Trypanosomatidae) transmitted to the vertebrate host by phlebotomine sand flies (Psychodidae: Phlebotominae). There are over 30 species of *Leishmania* in the New World, where *Leishmania enriettii* Muniz and Medina 1948 is an example of a non-infectious species to man [1]. Its vertebrate host is the guinea pig *Cavia porcellus*, and its suspected vector is *Lutzomyia monticola* [2,3].

Although non-infectious for humans, *Leishmania enriettii* is considered a model for the human cutaneous leishmaniasis (CL) [4-6]. An interesting aspect of this species is that it had never been found in any continent other than the Americas. However, kinetoplastid parasites from the *Leishmania enriettii* complex were recently found in infected Red Kangaroo (*Macropus rufus*) in Australia. In this context, the vector species was *Forcipomyia lasiohelea*, a ceratopogonid [7].

Glycoconjugates are very important during the interaction with the vertebrate and invertebrate hosts. In the New World species, the early events in the innate immune compartment are crucial for the development of a response against the parasite [8]. Glycoconjugates can be associated with glycosylphosphatidylinositol (GPI) anchors in the plasma membrane, especially the lipophosphoglycans (LPGs), gp63 and glycolinositolphospholipids (GIPLs) [9]. GIPLs are present on the cell surface in all stages and represent the most abundant glycoconjugates [9]. Structurally, GIPLs are widely polymorphic, but with a basic conserved structure of Manα1-4GlcN linked to the lipid portion, which usually consists of alkylacylglycerol or *lyso-*alkylglycerol (*lyso*-PI), by means of a phosphatidylinositol residue (PI) [10] and can be classified into three groups: Type I GIPLs have, for the greater part, a mannose residue as the most distal sugar, and can be identified by a substitution of the sixth carbon of the proximal mannose by a mannose residue (Manα1-6Manα1-4GlcN-PI). Type II GIPLs are characterised by the substitution of the third carbon of the proximal mannose by a mannose residue (Manα1-3Manα1-4GlcN-PI). The third group (hybrid GIPLs), shares structural features with the first two, possessing one additional mannose in the third and sixth carbons of the proximal mannose (Manα1-3(Manα1-6) Manα1-4GlcN-PI). Recently, *Leishmania infantum* and *Leishmania braziliensis* GIPLs were preliminarily characterised, as being of type I and Type II, respectively. An interesting feature of those molecules is their inhibitory properties via TLR4 during the interaction with BALB/c and C57BL/6 macrophages [11].

LPG is the most studied glycoconjugate in *Leishmania*. It is widely expressed in the surface of promastigote forms having four domains: a lipid anchor (1-O-alkyl-2- lysophosphatidylinositol); a heptasaccharide core (Gal(α1-6)Gal(α1-3)Gal_f_(α1-3)[Glcα1-PO_4_]Man(α1-3)Man(α1-4)-GlcN(α1-)); a region of repeat units (Gal(β1-4)Man(α1-)PO_4_) and the terminal neutral oligosaccharide cap [12]. LPG has been involved in a wide variety of functions including recognition, phagocytosis and protection from the acidic environment of parasitophorous vacuoles [13,14], resistance to complement, inhibition of phagosome maturation [15], inhibition of protein kinase C [16], induction of protein kinease R [17], the ability to intervene in the integrity of microdomains in phagosomal plasma membranes [18], modulation of nitric oxide (NO) and IL-12 production [16,19-21], modulation of MAPKs [21,22], agonist of TLR2 and TLR4 [17,21,23-25], induction of neutrophil extracellular traps (NETs) [26] and heme-oxigenase 1 [27] and attachment to the sand fly midgut [28-31].

Many studies have explored the role of *Leishmania* during the interaction with the vertebrate host [32]. However, most of those studies focused on Old World species of *Leishmania*. Recently, it was demonstrated that the LPG of *L. braziliensis* was more pro-inflammatory than that of *L. infantum*, suggesting that polymorphisms in the LPG structures may be important during the immunopathology of the disease. In C57BL/6 macrophages (and respective knock-outs) and CHO cells a predominant role of TLR2 was shown [21]. However, those and many other aspects are still unknown in *L. enriettii*.

In this work, we have studied two reference strains of *L. enriettii* isolated from *C. porcellus* in two distinct moments (1945 and 1985). This study aimed to preliminary characterise their glycoconjugates (LPG and GIPLs) and their role during *in vitro* interaction with macrophages and CHO cells. Additionally, their infectivity was tested with its natural vertebrate host in the presence and absence of salivary gland extract (SGE) from *Lutzomyia longipalpis*. This is part of a wider study on the glycobiology of New World species of *Leishmania*.

## Methods

### Ethics statement

All animals were handled in strict accordance with animal practice as defined by the Internal Ethics Committee in Animal Experimentation (CEUA) of Fundação Oswaldo Cruz (FIOCRUZ), Belo Horizonte (BH), Minas Gerais (MG), Brazil (Protocol p-0297-06). Knock-out mice handling protocol was approved by the National Commission of Biosafety (CTNBio) (protocol #01200.006193/2001-16).

### Parasites

World Health Organization Reference strains of *L. enriettii* (MCAV/BR/1945/L88 and MCAV/BR/1985/COBAIA_SP) were used. Promastigotes were cultured in M199 medium supplemented with 10% heat inactivated fetal bovine serum (FBS), penicillin 100 units/mL, streptomycin 50 μg/mL, 12.5 mM glutamine, 0.1 M adenine, 0.0005% hemin, and 40 mM Hepes, pH 7.4 at 26°C until late log phase [33].

### Extraction and purification of LPGs and GIPLs

**Figure 1:**
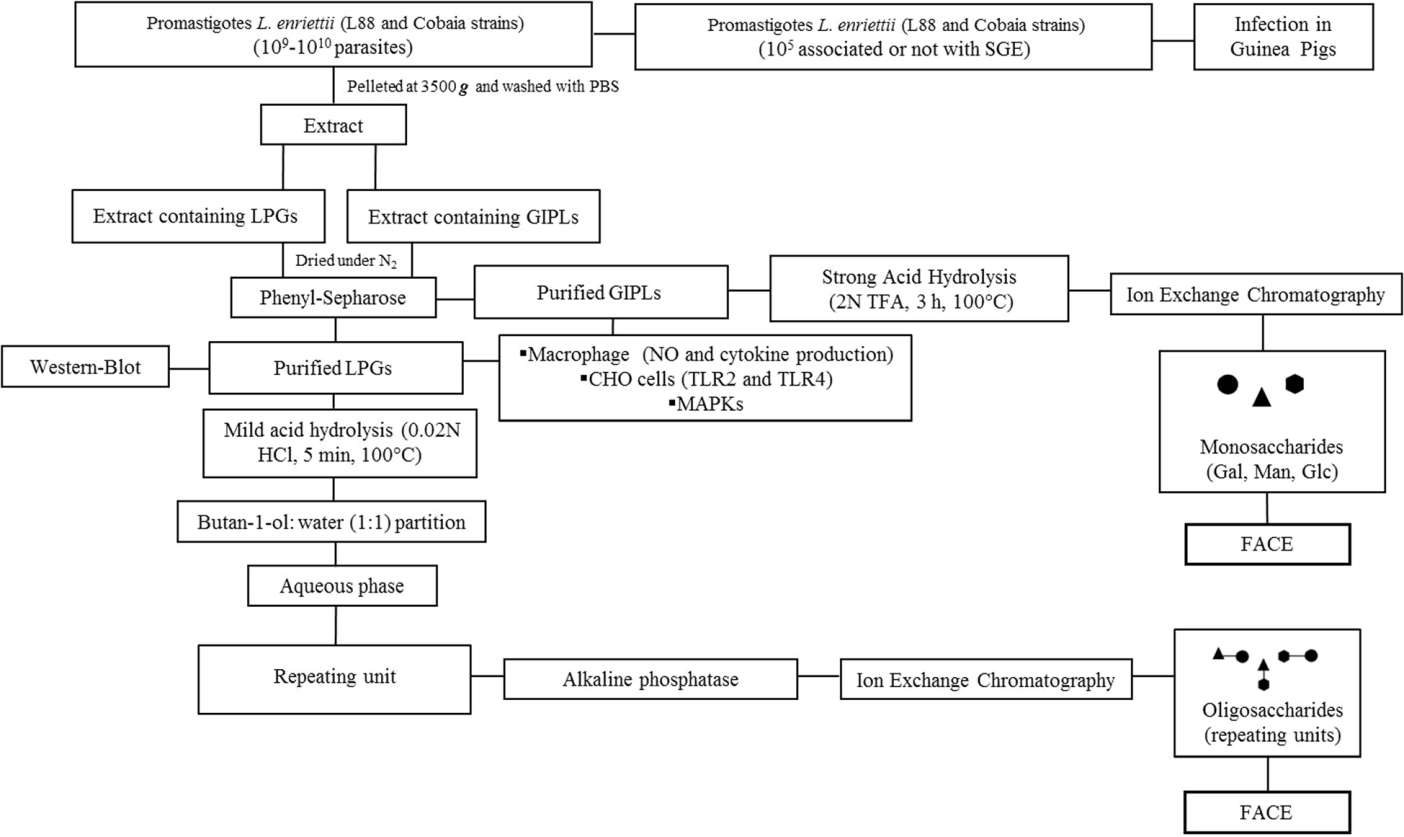
**Experimental procedures scheme.** Promastigotes of *L. enriettii* were used for infection in *C. porcellus* in the presence/absence of Salivary Gland Extract (SGE). LPG and GIPLs were extracted with organic solvents and purified using Phenyl-Sepharose. LPG purification was confirmed by western-blot. The LPG was depolymerased using mild acid hydrolysis (0.02 N HCl, 5 min, 100°C) and the repeat units were dephosphorylated using alkaline phosphatase. The profiles were analysed by Fluorophore-assisted carbohydrate electrophoresis (FACE). Purified GIPLs were subjected to strong acid hydrolysis (2 N trifluoroacetic acid, 100°C, 3 hours) and monosaccharides were analyzed by FACE. Purified LPGs and GIPLs were incubated with murine peritoneal macrophages, CHO cells and J774.A1 cells for NO, cytokines, MAPKs and NF-kB activation.

For optimal LPGs and GIPLs extractions, late log phase cells were harvested and washed twice with PBS prior to extraction of LPGs and GIPLs. The LPGs and GIPLs extractions were performed as described elsewhere with solvent E (H_2_O/ethanol/diethylether/pyridine/NH_4_OH; 15:15:5:1:0.017) after a sequential organic solvent extraction [12]. For purification, the solvent E extract was dried under N_2_ evaporation, resuspended in 2 mL of 0.1 N acetic acid/0.1 M NaCl, and applied onto a column with 2 mL of phenyl-Sepharose, equilibrated in the same buffer. The column was washed with 6 ml of 0.1 N acetic acid/ 0.1 M NaCl, then 1 mL of 0.1 N acetic acid and finally 1 mL of endotoxin free water. The LPGs and GIPLs were eluted with 4 mL of solvent E, dried under N_2_ evaporation and quantitated as described [34]. Prior to use on *in vitro* macrophage cultures, LPG and GIPLs were diluted in fresh RPMI. All solutions were prepared in sterile, LPS-free distilled water (Sanobiol, Campinas, Brazil). All experimental procedures are depicted in (Figure 1).

### Fluorophore-assisted carbohydrate electrophoresis (FACE)

#### Oligosaccharides

Phosphorylated repeat units were treated with alkaline phosphatase in 15 mM Tris buffer, pH 9.0 (1 U, 16 h, 37°C) (Figure 1). Samples were desalted and subjected to fluorophore-assisted carbohydrate electrophoresis (FACE). Samples were fluorescently labelled with 0.05 N ANTS (8-aminonaphthalene-1,3,6-trisulfate) and 1 M cyanoborohydride (37°C, 16 h). Oligoglucose ladders (G_1_–G_7_) were used as standards. Sugars were subjected to FACE and the gel was visualised by an UV imager [35].

#### Monosaccharides

To access the monosaccharide composition, deaminated GIPLs headgroups were subjected to strong acid hydrolysis (2 N trifluoroacetic acid, 3 h, 100°C) (Figure 1). To determine the monosaccharide composition of the GIPLs, depolymerised and desalted monosaccharides were fluorescently labeled with 0.1 M AMAC (2-aminoacridone) in 5% acetic acid and 1 M cyanoborohydride. Labeled sugars were subjected to FACE and the gel was visualized under UV light. Monosaccharides (D-galactose, D-glucose and D-mannose) (Sigma) were used as standards [11].

### Purification of murine peritoneal macrophages and cell culture

Thioglycollate-elicited peritoneal macrophages were extracted from C57BL/6 and C57BL/6 (TLR2−/− and TLR4−/− knockouts) by peritoneal washing with ice cold serum-free RPMI and enriched by plastic adherence for 1 h at 37°C/5% CO_2_. Cells (3x10^5^cells/well) were washed with fresh RPMI and cultured in RPMI, 2 mM glutamine, 50 U/mL of penicillin and 50 μg/mL streptomycin supplemented with 10% FBS in 96-well culture plates (37°C/5% CO_2_). Cells were primed with gamma interferon (IFN-γ) (3 IU/mL) [36] for 18 h prior to incubation with *L. enriettii* LPGs (10 μg/mL), GIPLs (10 μg/mL), *L. braziliensis* LPG (10 μg/mL), lipopolysaccharide (LPS) (100 ng/mL) or live stationary *L. enriettii* parasites (MOI 10:1).

### Cytokine and nitrite measurements

For CBA multiplex cytokine detection, cells were plated, primed as described above and incubated with LPGs, GIPLs, LPS and live stationary promastigotes (MOI 10:1) for 48 h. *Leishmania braziliensis* LPG (10 μg/mL) was added as a positive control [21]. For negative controls fresh medium was added. Supernatants were collected and IL1-β, IL-6, IL-10, IL-12p40 and TNF-α were determined using the BD CBA Mouse Cytokine assay kits according to the manufacturer’s specifications (BD Biosciences, CA, USA). Flow cytometry measurements were performed on a FACS Calibur flow cytometer (Becton Dickinson, Mountain View, CA). Cell-Quest™ software package provided by the manufacturer was used for data acquisition and the FlowJo software 7.6.4 (Tree Star Inc., Ashland, OR, USA) was used for data analysis. A total of 2,400 events were acquired for each preparation. Results are representative of two experiments in duplicate. Nitrite concentrations were determined by Griess reaction [21].

### Chinese Hamster Ovary (CHO) cell lines

The CHO reporter cell lines TLR2-TLR4-, which do not express TLR2 nor TLR4; TLR2+, expressing TLR2 and TLR4+, expressing TLR4 [37] were maintained as adherent monolayers in Ham’s F-12/DMEM supplemented with 5% FBS, at 37°C/5% CO_2_, and antibiotics. All cell lines were derived from clone 3E10, that has been stably transfected with a reporter construct containing the structural gene for CD25 under the control of the human E-selectin promoter. This promoter contains a NF-kB binding site; CD25 surface expression is completely dependent upon NF-kB translocation to the cell nucleus [21,38]. In order to evaluate the activation of NF-kB by LPGs and GIPLs, CHO reporter cells were plated at a density of 1×10^5^cells/well in 24-well tissue culture dishes. The following day, either molecule or bacteria (*Staphylococcus aureus* [10^3^ bacteria/well], positive control of TLR2; LPS (200 ng/well), positive control of TLR4; or LPGs (0.2 μg or 0.02 μg/ well) and GIPLs (0.2 μg or 0.02 μg/ well) from the two *L. enriettii* strains were added for 18 h. LPGs from *L. braziliensis* and *L. infantum* were used as positive and negative controls, respectively [21]. The cells were harvested with trypsin-EDTA, washed with medium and PBS. Subsequently, 1 × 10^5^ were cells were stained with PE-labeled anti-CD25 (mouse mAb to human CD25, R-PE conjugate; Caltag Laboratories, Burlingame, CA) 1:200 in PBS, on ice, in the dark, for 30 min. After labeling, the cells were washed twice with the same buffer, resuspended in 1 mM sodium azide in PBS, and examined by flow cytometry (BD Biosciences, San Jose, CA) for the expression of surface CD25 as described [37]. Analyses were performed using Cell Quest software (BD Biosciences).

### Activation of MAPKs

We investigated whether LPGs and GIPLs from the two *L. enriettii* strains could modulate MAPKs activation. J774.1 macrophages were plated as above on 24 well tissue culture plates (3 × 10^6^/well) for 18 h prior to assay [21]. The cells were washed with warm RPMI and incubated with LPG and GIPLs from both strains for different times (5, 15, 30 and 45 min) or with medium (negative control) or LPS (100 ng/ml) as positive control. Cells were then washed with ice-cold PBS and lysed in RIPA lysis buffer (Sigma) and protease inhibitor cocktail (Thermo Scientific). Cells were harvested with a plastic scraper and centrifuged at 13,000 × g (4°C, 10 min). Supernatants were transferred to new tubes and stored at −20°C until used for immunoblotting. Cell lysates were resolved by SDS-PAGE, transferred to a nitrocellulose membrane and blocked (5% milk in TBS-0.1% Tween 20) for 1 h. Primary Abs [dually phosphorylated p38 (Santa Cruz) and JNK (Sigma) 1:1,000, total p38 primary antibody (Sigma) was used as a normalizer] were incubated for 16 h at 4°C. Membranes were washed (3 × 10 min) with TBS-0.1% Tween 20 and incubated 1 h with anti-mouse IgG conjugated with peroxidase (1:10,000). The reaction was visualised using luminol [21]. The data were analysed by Densitometry using the software ImageJ 1.48v by National Institutes of Health (http://imagej.nih.gov/ij).

### Sand flies

*Lutzomyia longipalpis* sand flies were captured in Teresina, Piauí state, Brazil. The insects were reared in the Laboratório de Fisiologia de Insetos Hematófagos at the Universidade Federal de Minas Gerais in Brazil using existing methodology [39]. Three to 6-day-old non-fed female sand flies, maintained on 30% sucrose were dissected in PBS. Heads, crops, hindguts, and Malpighian tubules were removed, and the isolated salivary glands were dissected. Ten salivary glands were dissected, placed in 20 μL of PBS and stored at -80°C. Immediately before use, the glands were sonicated for 10 seconds in a water bath [40] and centrifuged (2100 ***g***, 10 min). The supernatant containing Salivary Gland Extract (SGE) was mixed with 1 × 10^6^ parasites/ml in PBS prior to *in vivo* experiments.

### *In vivo* infections with guinea pigs

For intradermal inoculation, we used 1×10^5^*L. enriettii* promastigotes in a volume of 100 μl in PBS [40], associated or not with SGE. Forty males of *Cavia porcellus* (20 per experiment) were divided into 4 groups: group 1 (infected with L88 strain), group 2 (infected with L88 strain + SGE of *L. longipalpis*), group 3 (infected with Cobaia strain) and group 4 (infected with Cobaia strain + SGE of *L. longipalpis*). The course of infection was assessed weekly for 91 days by measuring the lesion areas (mm^2^).

### Statistical analyses

For nitrite and cytokine measurements, the Shapiro Wilk test was conducted to test the null hypothesis that data were sampled from a Gaussian distribution (SHAPIRO, 1965). The P value (P > 0.05) showed that data did not deviate from Gaussian distribution. For this reason, Student’s “t” test and ANOVA were performed to test equality of population medians among groups and independent samples. Data were analyzed using GraphPad Prism 5.0 software (Graph Prism Inc., San Diego, CA) and P < 0.05 was considered significant.

## Results

### Growth curve

A growth curve was established to determine the division profile of the parasites for glycoconjugate extraction. The strains showed similar growth patterns reaching the stationary phase after the 10^th^ day. Cobaia reached higher densities than L88 (8.7×10^6^ versus 6.5 × 10^6^ parasites/mL) (P < 0.05). For this reason, we selected the 10^th^ day of growth for LPG and GIPLs extraction for both strains.

### LPG purification

**Figure 2:**
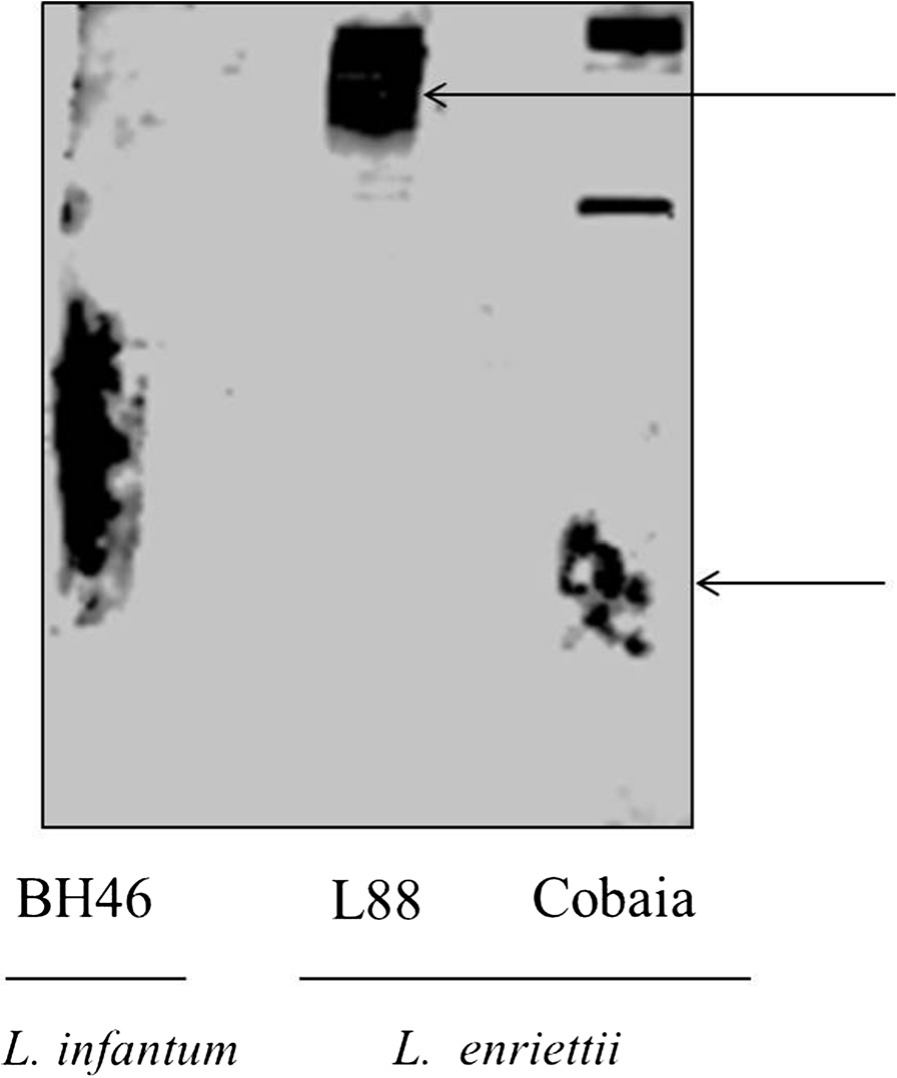
**Western blot of purified LPG (10 μg per lane) of** ***L. infantum*** **(BH46 strain) and** ***L. enriettii*** **(L88 and Cobaia strains) in the presence of CA7AE antibody (1:1000).** Upper and lower arrow indicates the smears of *L. enriettii* LPGs (L88 and Cobaia strains), respectively.

In Western Blots, purified LPGs from both *L. enriettii* strains were recognized by the antibody CA7AE, whose structural epitope is the unbranched Gal(β1,4)Man(α1-PO_4_) repeat units, common to all LPGs [41]. The LPG of the L88 strain showed a higher molecular weight smear (upper arrow) in Western Blots than that of the Cobaia strain (lower arrow), suggesting that the former possessed a longer LPG. As expected, the positive control (LPG of the BH46 *L. infantum* strain) [42] was recognised by the antibody (Figure 2).

### Profile analysis of the LPG repeat units

**Figure 3:**
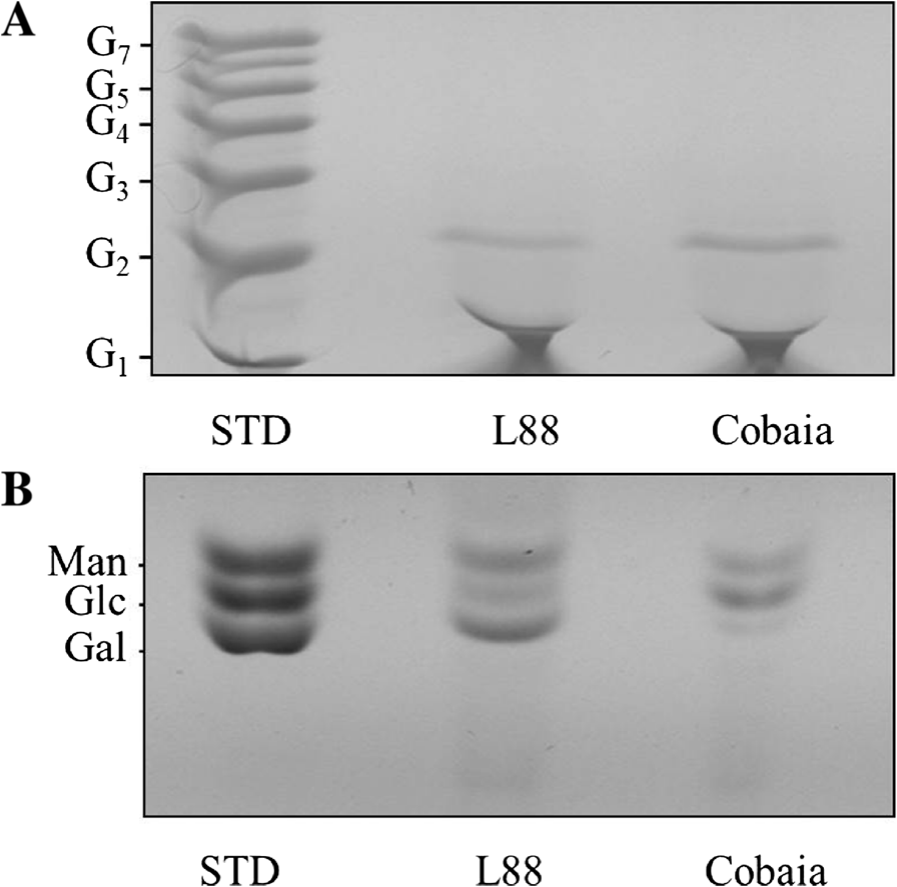
**Carbohydrate profile of LPGs (repeat units) and GIPLs (core) from** ***L. enriettii*** **(L88 and Cobaia strains).** STD, standard represented by oligoglucose ladder (G_1_-G_7_). **(A)** Oligosaccharide profile from LPG repeat units. **(B)** Monosaccharide profile of *L. enriettii* (L88 and Cobaia strains). STD, standard represented by the monosaccharide sugars (100 μg/mL). Man, mannose, Glc, glucose, Gal, galactose.

All LPGs were subjected to mild acid hydrolysis to obtain their repeat units (Figure 1). These units were tagged with a fluorescent probe at the reducing ends and subjected to FACE for visualisation of the carbohydrate profiles. In order to identify the number of sugars, we used a standard molecular weight of oligoglucoses (G_1_-G_7_), whose bands indicate the migration profile from mono to heptasaccharides (Figure 3A, STD lane). Both strains showed a band at the G_2_ position, an expected result since all LPGs possess the Gal-Man disaccharide (Figure 3A). Therefore, these data indicate that the LPGs from both *L. enriettii* strains are devoid of side chains.

### Identification of GIPL monosaccharides

In order to determine the monosaccharide composition of GIPLs, the purified GIPLs were subjected to strong acid hydrolysis (Figure 1B). Unlike what was observed in the polysaccharide gel (Figure 3A), there was a polymorphism in the GIPLs composition. As expected, mannose and galactose are present in both strains of *L. enriettii*. The L88 strain is characterised by being rich in the monosaccharide galactose, suggesting similarity with the type II GIPL [11]. On the other hand, the structure of the GIPL from the Cobaia strain contains the sugars mannose and glucose in its composition. It is noteworthy that this GIPL contains glucose, a feature never observed in other *Leishmania* GIPLs (Figure 3B).

### Analysis of NO production in murine macrophages

**Figure 4:**
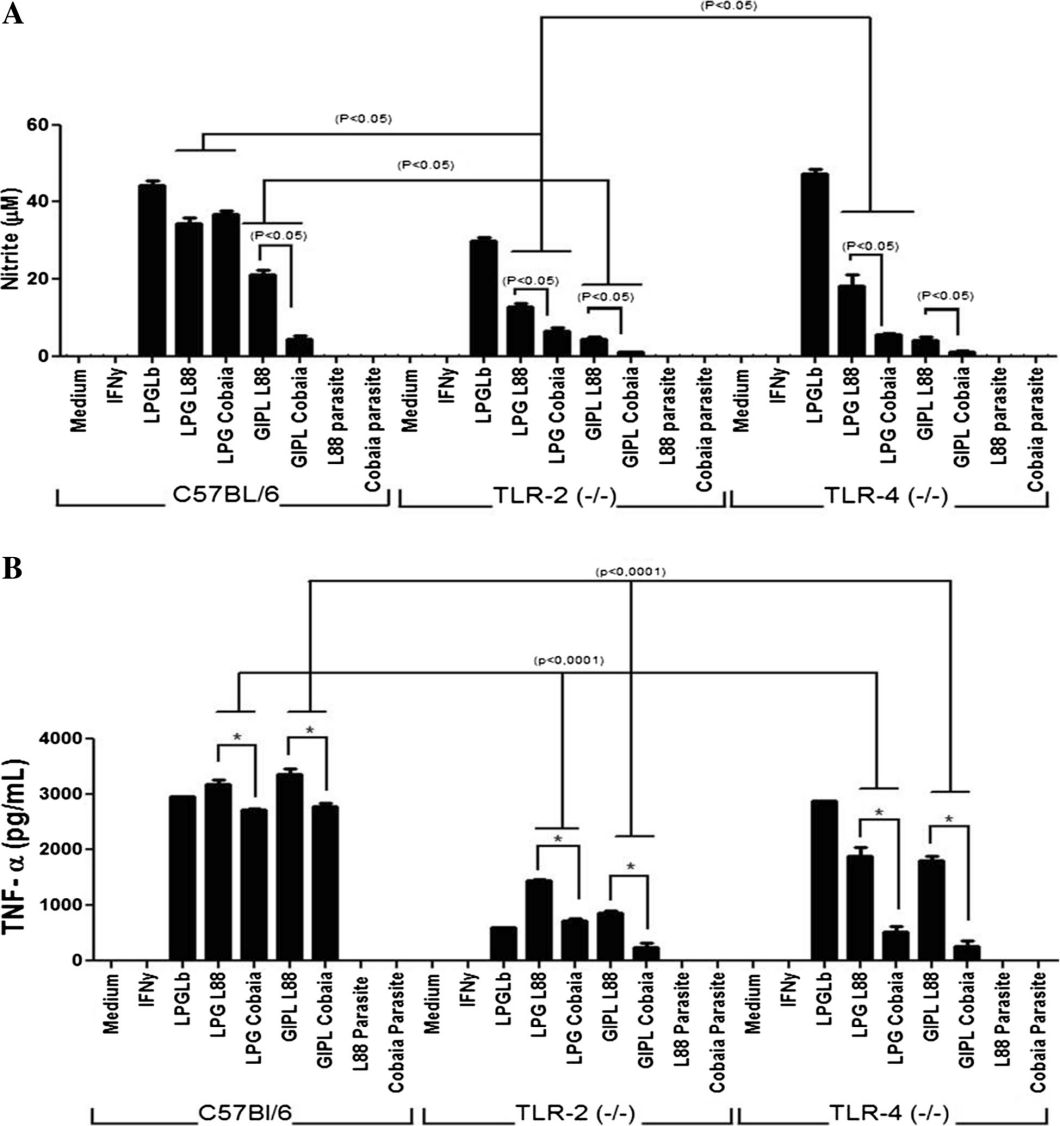
**Nitrite and TNF-α production by IFN-γ-primed macrophages after stimulation with LPGs (10 μg/mL), GIPLs (10 μg/mL) and parasites. (A)** NO concentrations were measured by Griess reaction. **(B)** TNF-α concentrations were determined by flow cytometry. ANOVA test was performed and P < 0.05 was considered significant. The results represent the average of two experiments in duplicate. LPGLb, *L. braziliensis* LPG.

In order to determine the NO production profile in peritoneal macrophages of the C57BL/6, TLR2 (-/-) and TLR4 (-/-) lineages, and possible participation of TLRs, these cells were stimulated with LPGs, GIPLs, LPS and promastigote forms from both *L. enriettii* strains. No activation was observed for the living parasites, growth medium or IFN-γ (Figure 4A). There was no statistical difference regarding NO synthesis in the wild mice stimulated with LPGs from both strains. However, a higher NO production was observed in macrophages incubated with LPGs (Knock-outs) and GIPLs (all lineages) from L88 strain (P < 0.05). This activation was primarily via TLR2, and secondarily via TLR4 (P < 0.0001) (Figure 4A).

### Analysis of cytokine production in murine macrophages

**Figure 5:**
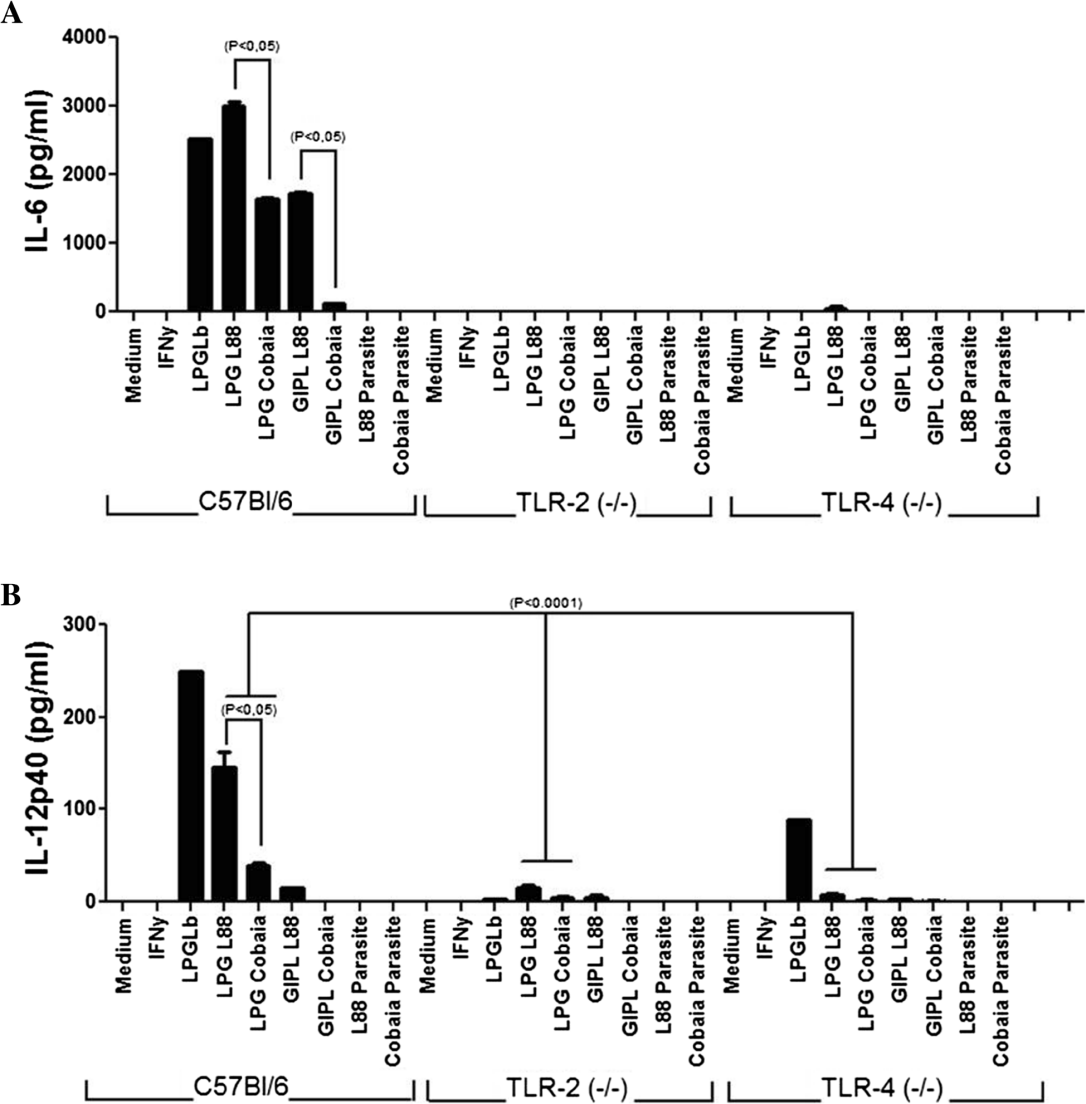
**IL-6 and IL12p40 production by IFN-γ-primed macrophages after stimulation with LPGs (10 μg/mL), GIPLs (10 μg/mL) and parasites. (A)** IL-6 and **(B)** IL-12p40 concentrations were determined by flow cytometry. ANOVA test was performed and P < 0.05 was considered significant. The results represent the average of two experiments in duplicate. LPGLb, *L. braziliensis* LPG.

Similarly to the NO experiment, supernatants from macrophage cultures of the three strains of mice (C57BL/6, TLR2 (-/-) and TLR4 (-/-)) were subjected to CBA analysis to quantitate the cytokines TNF-α, IL-1β, IL-6, IL-10 and IL-12p40. Similar to previous results with other *Leishmania* species [11,21], no cytokine production by living parasites from both strains was observed (Figure 4B; Figure 5). A higher pro-inflammatory activity was observed for the LPG and GIPL from L88 strain, where higher amounts (pg/ml) of the citokines TNF-α, IL-6 and IL-12p40 were detected (P < 0.05). Similarly to NO, this production was primarily via TLR2 than TLR4, especially for TNF-α (Figure 4B). No significant levels were detected for the cytokines IL-10 and IL-1β in any of the experiments (data not shown).

### Analysis of NF-kB translocation by LPGs and GIPLs

**Figure 6:**
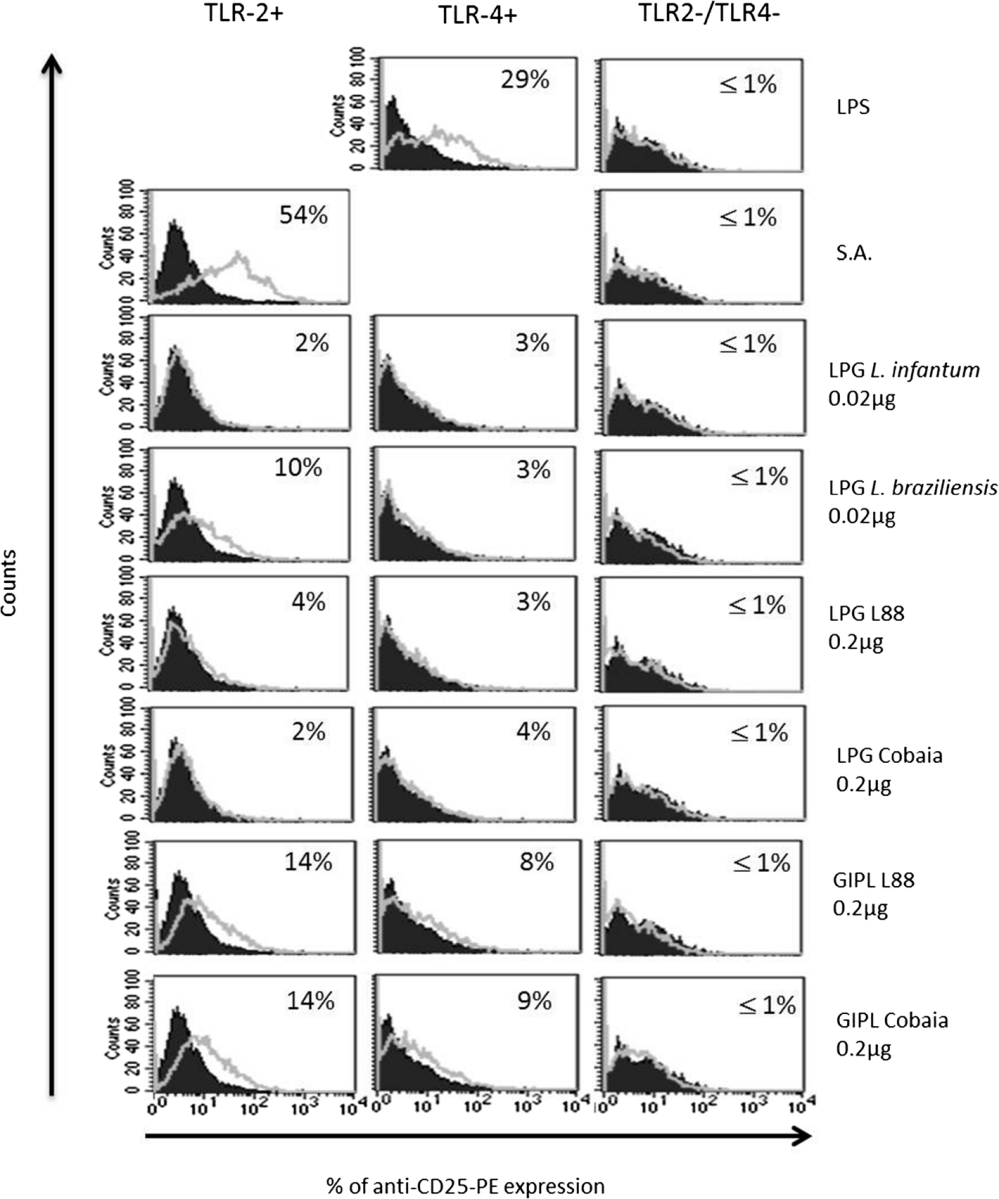
**GIPLs purified from two strains of** ***L. enriettii*** **(L88 and Cobaia) induce translocation of NF-kB through TLRs.** CHO cells expressing TLR2 (TLR2+), TLR4 (TLR4+), or neither (TLR2-/TLR4-) were either untreated (black line) or exposed (gray line) to LPS, *S. aureus* (SA), *L. infantum* LPG, *L. braziliensis* LPG, *L. enriettii* strain L88 LPG (LPG L88), *L. enrietti* strain Cobaia LPG (LPG Cobaia), *L. enriettii* strain L88 GIPL (GIPL L88) or *L. enriettii* strain Cobaia GIPL (GIPL Cobaia), as indicated. CD25 expression was measured by flow cytometry 18 h after stimulation. Percentage = percentage of CD25 expression on stimulated cells minus percentage of CD25 expression on non-stimulated cells.

Considering that TLR2 and TLR4 were the main receptors recognising the LPGs and GIPLs from both *L. enriettii* strains, the role of each of these receptors was assessed separately in CHO cells. These cells were treated with LPGs and GIPLs for 18 hours and the expression of the CD25 reporter was analysed by flow cytometry [21]. No activation of TLR2 and TLR4 receptors was observed after incubation with LPG (0.2 and 0.02 μg). However, a higher activation of GIPLs, especially in TLR2 than TLR4 was observed (Figure 6). As expected, the positive control groups for TLR2, represented by the LPG of *L. braziliensis* and the lysate of *S. aureus*, activated the translocation of NF-kB [21]. The positive control group for TLR4, represented by LPS, also activated NF-kB. Negative control represented by LPG of *L. infantum* (BH46 strain) [21], did not activate NF-kB (Figure 6).

### Activation of MAPKs

**Figure 7:**
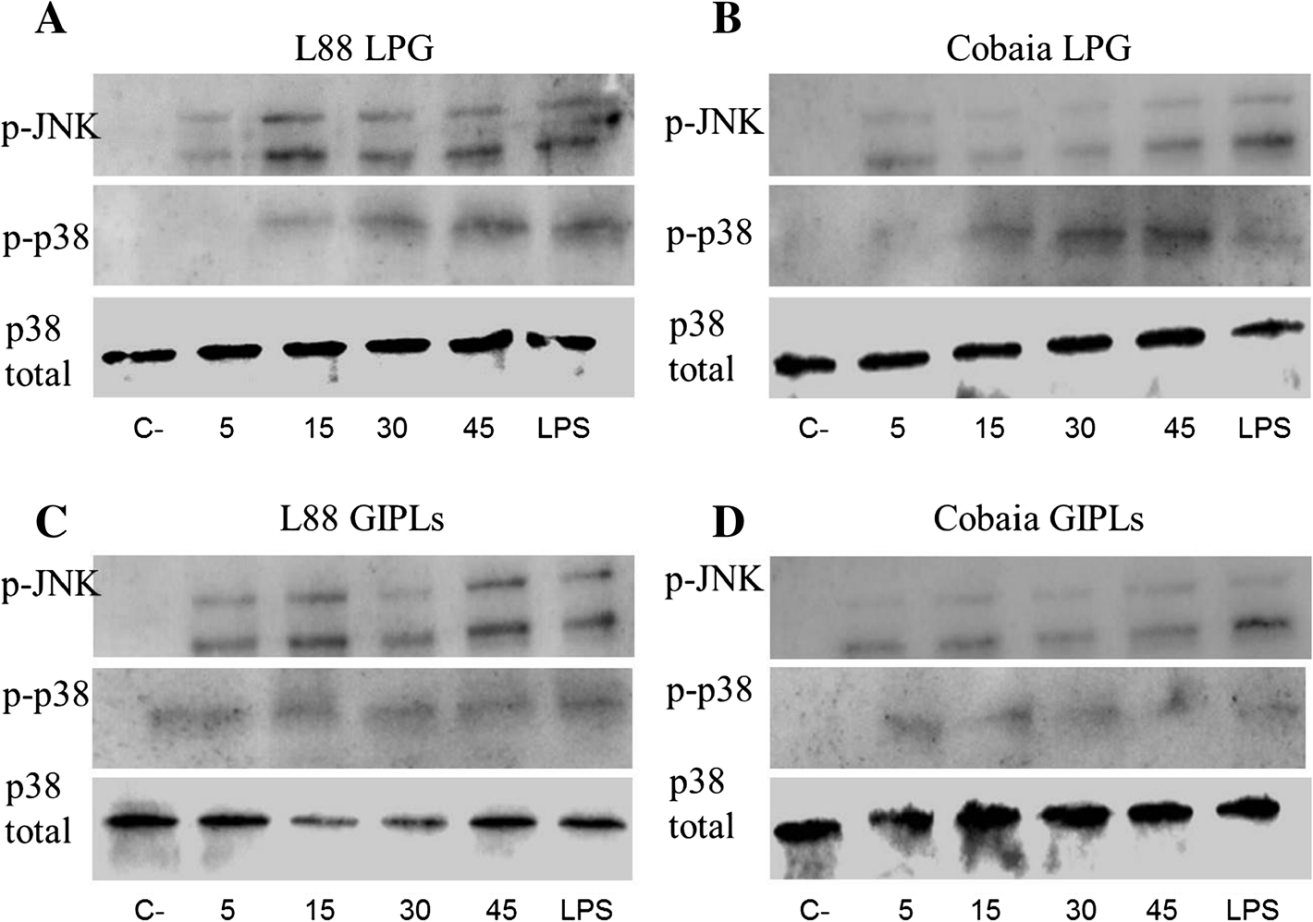
**Activation of MAPKs (p38 and JNK) by** ***L. enriettii*** **glycoconjugates (LPG and GIPLs) from both strains (L88 and Cobaia) in J774A.1 peritoneal macrophages.** Macrophages were stimulated for 5, 15, 30 and 45 min with 10 μg/mL of LPG and GIPLs from *L. enriettii* L88 **(A, C)** and *L. enriettii* Cobaia **(B and D)**. Dually phosphorylated MAPKs (JNK and p38) were detected by western blot. C-, negative control; Total p38 content was used as the normalizing protein.

To better access the signaling events around LPGs and GIPLs recognition and macrophage activation, J774.1 macrophages were incubated with *L. enriettii* (L88 and Cobaia strains) LPGs and GIPLs. MAPK activation was assessed as a function of time and analysed by Densitometry. Similarly to cytokine production, the LPG of L88 strain was able to activate JNK more pronounced than Cobaia strain (Figure 7A and B). No differences in p38 activation were observed for LPG and GIPLs between the two strains (Figure 7C and D).

### Infection in *Cavia porcellus* using the two *L. enriettii* strains

Since glycoconjugates (LPG and GIPLs) from the two *L. enriettii* strains exhibited a distinct pro-inflammatory profile during *in vitro* experiments, live parasites were inoculated in *C. porcellus* for evaluation of their infectivity.

**Figure 8:**
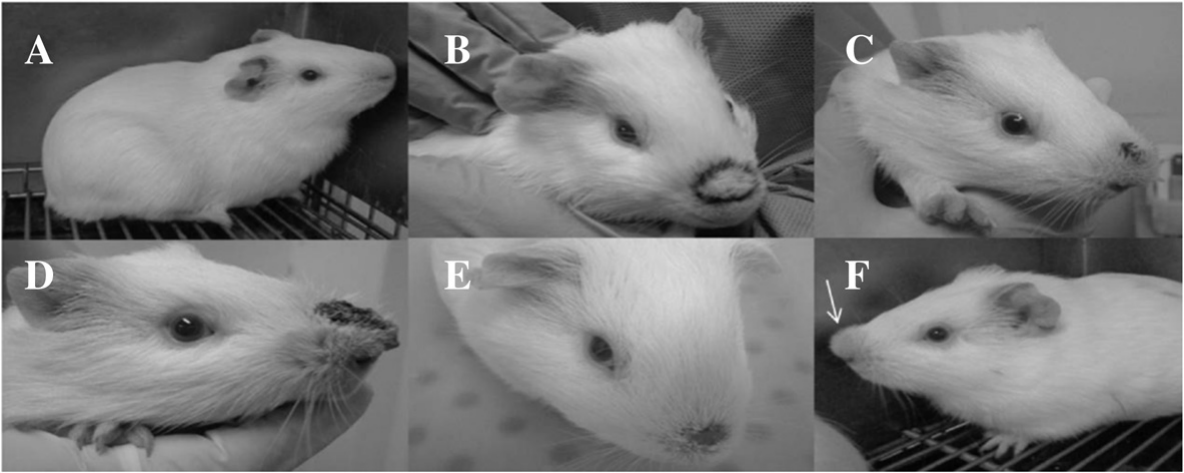
**Lesions in** ***C. porcellus*** **infected by** ***L. enriettii*** **strains in the presence/absence of salivary gland extract (SGE) from** ***Lutzomyia longipalpis.*** Male *C. porcellus* were inoculated with 1x10^5^ parasites of *L. enriettii* strains (L88 and Cobaia). **(A)** non-infected *C. porcellus*; **(B)** *C. porcellus* infected with L88 strain (4 weeks of infection); **(C)** *C. porcellus* infected with L88 strain (5 weeks infection); **(D)** *C. porcellus* infected with L88 strain + SGE (7 weeks of infection); **(E)** *C. porcellus* infected with L88 strain (8 weeks of infection) and **(F)** *C. porcellus* infected with Cobaia strain (4 weeks of infection).

**Figure 9:**
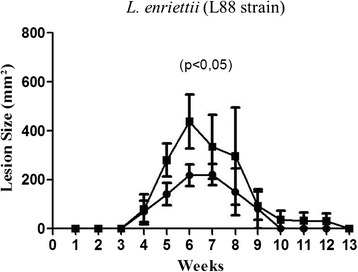
**Development of lesions (mm** ^**2**^ **) in** ***C. porcellus*** **after inoculation with** ***L. enriettii*** **(L88 strain) in the presence/absence of salivary gland extract (SGE) from** ***L. longipalpisv.*** Dark circles, *C. porcellus* infected with *L. enriettii* strain L88 and dark squares, *C. porcellus* infected with *L. enriettii* strain L88 + SGE. Male *C. porcellus* were inoculated with 1x10^5^ parasites of *L. enriettii* (L88 strain). Lesion size (mm^2^) was followed weekly.

No mortality was observed during the experiment (91 days). There was no statistical difference between the weights of groups 1 and 2, and between groups 3 and 4 throughout the experiment (P > 0.05). In spite of the absence of lesions, all Cobaia infected animals exhibited protuberances (Figure 8F). For this reason, lesion analysis was restricted to L88 strain (Figures 8A-E and Figure 9). Infected animals started developing protuberances and/or lesions during the fourth-fifth week of infection (Figure 9). The presence of SGE resulted in an increase of lesion size (477 ± 220.2 versus 294.2 ± 120.0 mm^2^) (P < 0.05). Also, a greater onset-healing period (ten weeks) was observed for SGE injected animals. After this period, a gradual decrease in the area of the lesion was noticed with a complete absence of lesion after the thirteenth week (Figure 9).

## Discussion

This work compared two *L. enriettii* strains isolated from the wild between a 40-year interval*.* The parameters studied included: growth curve, biochemical analysis of glycoconjugates (LPGs and GIPLs), NO and cytokine production, receptors involved, signaling pathways and *in vivo* infectivity for their natural host. This study aimed to enhance our understanding of molecular aspects of *L. enriettii* biology and glycobiology.

The two *L. enriettii* strains showed a lower growth pattern in M199 medium in comparison to *L. infantum* and *L. braziliensis* from previous studies under the same conditions, never reaching a density above to 1 × 10^7^ cells/mL [33,43]. Higher densities were observed for the Cobaia strain, although both achieved stationary phase at the tenth day of culture. This indicates that the strains possess different division profiles.

**Figure 10:**
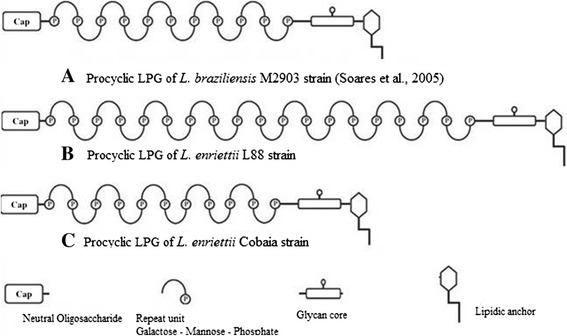
**Schematic diagram of** ***L. enriettii*** **LPGs.** The central portion of the structure is Gal(α1,6)Gal(α1,3)Galf(α1,3)[Glc(α1-PO_4_)-6]Man(α1,3)Man(α1,4)GlcN(α1:6), attached lipid anchor to alkyl-2-lyso-1-*O* phosphatydylinositol (PI). The repeat units are 6-Gal(β1,4)Man(α1)-PO_4_. The precise numbers of the repeat units in the * L. enriettii* LPGs and the CAP constitution are not known. P = phosphate.

Studies focusing on *Leishmania*-host interaction are important for understanding parasite biology. Surface glycoconjugates are key factors during this process. They enable successful infection in the hostile environments present both in the vertebrate and invertebrate hosts [8,44]. The LPG is the most studied *Leishmania* glycoconjugate not only in the Old World [9,35,45-47], but also in the New World [8,21,33,42,43,48]. The LPGs of *L. mexicana* [48] and *L. infantum* (PP75 strain) have one β-glucose linked to the repeat units. In *L. infantum*, this β-glucose is down-regulated in expression after metacyclogenesis [33]. The opposite happens with *L. braziliensis*, where the procyclic forms do not have side chains and the metacyclic forms have 1-2 β-glucoses [43]. In this study, the LPG and preliminary GIPL structures of *L. enriettii* were determined. Based on our data, the LPGs were devoid of side chains, being represented by the disaccharide Gal-Man common to all LPGs. The LPGs from the two *L. enriettii* strains were similar to the LPGs of *L. braziliensis* (strain M2903), *L. infantum* type I and *L. donovani* (Sudan strain) [42,43,46]*.* A distinguished feature of *L. enriettii* strain L88 was observed in the Western-Blot. Its higher molecular weight is consistent with a longer disaccharide chain as detected by the antibody CA7AE, specific for the Gal-Man repeat units of the LPG [41]. Previous studies with *L. major* and *L. infantum* have shown that more complex LPG structures were able to trigger a higher NO production by macrophages [20,42]. Consistent, with those data, the longer LPG from *L. enriettii* (L88 strain) exhibited a greater ability to induce NO production by macrophages. The preliminary structures of *L. enriettii* LPGs are depicted in Figure 10.

*Leishmania* GIPLs may be classified as type I, II and hybrids [revised by 8]. Type I GIPLs (mannose rich) are found in *L. infantum* [11], *L. donovani*, *L. tropica* and *L. aethiopica* [45]. The galactose-rich type II GIPLs are commonly found in *L. braziliensis* [11], *L. major* [10], *L. mexicana* [9] and *L. panamensis* [47]. Finally, hybrid GIPLs have mixed structural features of type I and II. It is found in *L. mexicana* and *L. donovani* [9]. Different from LPGs, the GIPLs from the two strains exhibited polymorphisms in their sugar compositions. The L88 strain was galactose rich, suggesting its similarity to a type II GIPL. On the other hand, Cobaia strain had a composition rich in glucose, a feature never previously observed in any GIPL of *Leishmania* [11].

The polymorphisms observed and the LPG size and in the GIPL composition from both strains were evaluated *in vitro* using murine peritoneal macrophages in order to establish their pro-inflammatory properties in the innate immune compartment. Some of those mechanisms were observed in this work during interaction with *L. enriettii*.

In *Leishmania*, TLR2, TLR4 and TLR9 are the main receptors activated by parasite PAMPs [8,32,49,50]. Their activation is dependent on MyD88 adapter protein resulting in the production of nitric oxide and pro-inflammatory cytokines [21,23,24]. Previous studies have shown that *Leishmania* LPGs and GIPLs are agonists of TLR2 and TLR4, respectively [11,21,23,24]. In *L. enriettii* glycoconjugates, a differential stimulation pattern was observed by those molecules in murine macrophages, suggesting the participation of both TLR2 and TLR4 by murine macrophages for both strains. Interestingly, a more pro-inflammatory activity was observed for L88 strain in comparison to Cobaia strain not only for LPG, but also for GIPLs. Previous studies using *L. major*, *L. braziliensis* and *L. infantum* GIPLs have shown the anti-inflammatory function of this glycoconjugate thus inhibiting the NO production when stimulated with IFN-γ and LPS [11,51]. This profile was not observed in this study, where the two *L. enriettii* strains were able to trigger the production of NO and other cytokines. In conclusion, the higher pro-inflammatory activity of the L88 LPG may be related to its size, whereas the higher pro-inflammatory activity of their GIPLs may be due to the presence of a greater galactose content. Consistent with those data, a similar profile was observed interspecifically with *L. braziliensis* and *L. infantum*. A higher pro-inflammatory activity was observed for *L. braziliensis* LPG and this was due to their ability to translocate NF-kB [21]. Consistent with those observations, JNK activation was more pronounced in L88 LPG than Cobaia LPG. However, we could not detect any substantial difference for p38 after stimulation with LPG and GIPLs from both strains. Confirming this pattern, the ability to activate p38 was also observed for *L. donovani* LPG in J774A.1 macrophages [52]. However, *L. braziliensis* and *L. infantum* GIPLs were not able to activate murine macrophages [11], a strong indication that this modulation may be species specific.

In the present study, the activation of NO and cytokines by the LPGs and GIPLs of the two *L. enriettii* strains was done preferably via TLR2 and secondarily via TLR4. The GIPLs data differ from those observed in *L. major* [51] and *L. braziliensis*/*L. infantum* [11], whose activation was mainly through TLR4. Those features are consistent with the diversity observed in *Leishmania* species, whose variations in their glycoconjugates may account at least in part for those differences. Since a dual role of TLR2 and TLR4 was observed after stimulation with both glycoconjugates, the next step was to observe their individual role using CHO cells.

The individual evaluation of the function of each TLR utilizing CHO cells, showed that the LPGs were not able to activate TLR2 and TLR4 and this feature is very similar to that observed for *L. infantum* LPG [11]. This is the first time that GIPLs were exposed to CHO cells. Consistent with our previous results in C57BL/6 mice, the GIPLs were able to activate either TLR2 or TLR4. The fact, that LPGs were not able to separately activate CHO transfected cells may suggest a co-participation of the two receptors by *L. enriettii* LPGs. It is well known that for lipopeptides of bacteria and GPI-mucins of *T. cruzi* [53], a dimerization between TLR1/6 and TLR2/6 is reported [54]. However, the phenomenon of dimerization between TLR2/4 should be further explored in *Leishmania*.

Based on the distinguished profile observed in the pro-inflammatory studies, we examined the infectivity of both strains in its natural host *C. porcellus*. Previous studies have shown that small amounts of sand fly saliva could promote the infection when inoculated with promastigotes of *Leishmania* spp [40,55-57]. Consistent with those data, in the animals infected with L88 strain plus SGE, the lesion size was 38.35% larger and took longer to heal. On the other hand, those infected with the Cobaia strain did not develop ulcerated lesion. Those data confirmed the potential of the *L. longipalpis* saliva in exacerbate *Leishmania* infection and the more pro-inflammatory activity of L88 strain which was the only one able to develop lesions in *C. porcellus* [58-62].

## Conclusions

In conclusion, our results showed that the strains of *L. enriettii* isolated in two distinct periods (1945 and 1985) differ biologically in the parameters studied. The *in vitro* pro-inflammatory profile and their infectivity *in vivo* were confirmed in its vertebrate host. A distinguished feature of *L. enriettii* glycoconjugates is that their LPGs could induce a higher production of IL-12 and their GIPLs were very pro-inflammatory. This is very different from what was reported in human pathogenic species such as *L. braziliensis* and *L. infantum* [8,11,21]. This may lead to the speculation that in the human, the excessive activation of the innate immune system by *L. enriettii* glycoconjugates may prevent this host to get infected with this parasite. This study is part of a wider project on the glycobiology of New World species of *Leishmania*.
